# Reply to ‘Comments on “Evidence of the hydrogen release mechanism in bulk MgH_**2**_”’

**DOI:** 10.1038/srep43720

**Published:** 2017-04-07

**Authors:** Kazuhiro Nogita, Xuan Q. Tran, Tomokazu Yamamoto, Eishi Tanaka, Stuart D. McDonald, Christopher M. Gourlay, Kazuhiro Yasuda, Syo Matsumura

**Affiliations:** 1 Nihon Superior Centre for the Manufacture of Electronic Materials, School of Mechanical and Mining Engineering, The University of Queensland, Brisbane, QLD 4072, Australia; 2 Department of Applied Quantum Physics and Nuclear Engineering and the Ultramicroscopy Research Center, Kyushu University, Fukuoka, 819-0395, Japan; 3 Department of Materials, Imperial College, London. SW7 2AZ. UK

## Abstract

In a comment on our Article “Evidence of the hydrogen release mechanism in bulk MgH_2_”, Surrey *et al*. assert that the MgH_2_ sample we studied was not MgH_2_ at any time but rather MgO; and that the transformation we observed was the formation of Kirkendall voids due to the outward diffusion of Mg. We address these issues in this reply.

The comment by Surrey *et. al.*^1^ on our recently published study^2^ contains two main assertions:

1. The MgH_2_ sample we studied was not MgH_2_ at any time but rather MgO.
2. The transformation we observed was the formation of Kirkendall voids due to the outward diffusion of Mg.

These are dealt with below.

## The presence of MgH_2_

It is possible that MgO [001] could have a good fit to the pattern compared with the MgH_2_ [101] zone axis. However, we are confident the diffraction pattern is from MgH_2_.

Firstly, MgO is most likely to exist in a nanocrystalline form on the outer surface of the bulk specimen under atmospheric exposure and hence, ring patterns would have been found in the SAD instead of diffraction spots (See for example Figure 2 of the comments by Surrey *et al*.)^1^.

**Figure 1:**
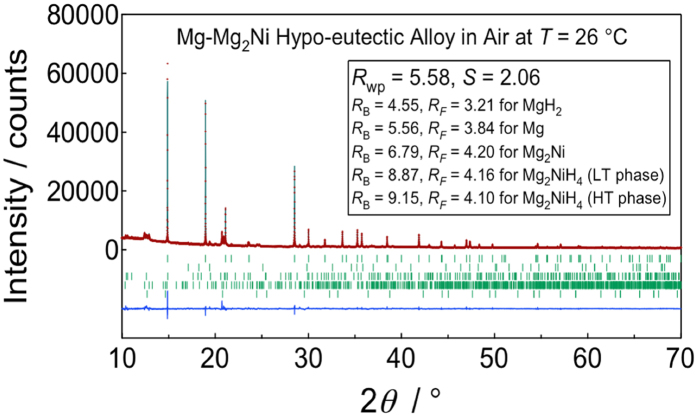
XRD peak profile and Rietveld refinement for sample tested under 0.1 MPa in-air, at 26 °C^3^ .

Furthermore the detailed crystallography of our sample can be found from the supporting Synchrotron XRD data which is discussed in the supplementary section of our paper (as Figure S3)^2^. This confirms that the as–prepared bulk sample contains mainly MgH_2_ and a small amount of other phases including Mg, Mg_2_Ni, Mg_2_NiH_4_ but not MgO. Refer to Fig. 1 below for a more detailed XRD peak profile and Rietveld refinement for a sample tested under 0.1 MPa in-air, at 26 °C^3^.

## Dehydrogenation of MgH_2_ or diffusion of Mg and Kirkendall void formation

We are confident we observed dehydrogenation in our samples using ultra-high voltage transmission electron microscopy (UHV-TEM). Firstly we have provided two additional independent measurements of dehydrogenation occurring at a temperature in the vicinity of 400 °C in the material we used. The first is based on synchrotron XRD as in Fig. S3 of our paper^2^ and the second on DSC as in Figure S5 of our paper^2^. For comparison, literature reported results for hydrogen desorption of MgH_2_ are in the range of 320–450 °C dependent on the presence of various catalysts (e.g. NaNH_2_^4^, Fe^5^, FeCl_3_^6^ or graphite^7^, respectively) and the heating rate. In the UHV-TEM the temperature range of the transformation we observed was between 400–455 °C, consistent within reason for the transformation of MgH_2_ to Mg.

The question that then remains is what was the difference between the samples and observation techniques used by Surrey *et al*.^1^ which made the MgH_2_ so unstable and the dehydriding reaction so fast as to make observation difficult ? The answer of course is that we studied large samples a few microns in size that were relatively free of deformation in UHV-TEM (1,000 kV), while Surrey *et al*.^1^ studied small 100 nm samples that were produced by high-energy ball-milling in low voltage TEM (300 kV).

The bulk samples we used were prepared by conventional casting and machining methods which contrast greatly with other severe deformation methods (e.g. ball milling). As a result, our sample could be handled under normal atmosphere without any significant oxidation as compared to materials processed by ball milling methods. In fact, several of our previous works have been carried out using this approach^2^,^8^,^9^. Reference 16 provided by Surrey *et al*.^1^ relates to their interpretation of our data that the transformation we observe is the formation of voids. This interesting publication shows this is a mechanism that operates in Mg particles of size 15–20 nm over a time period of hours but that Mg particles >50 nm are stable. The transformation in our sample takes place in a period of 10–20 minutes and, as the length scale is a few microns, it would seem to make this mechanism unlikely.

Secondly, conventional TEM with an accelerating voltage of 200 kV–300 kV has disadvantages including inelastic incident beam interactions with the samples, and the sample dimensions (typically less than 100 nm in thickness) make surface effects more prominent^10^, which becomes particularly important in small samples with a high specific surface area. In this aspect, UHV-TEM is more favorable for determining the mechanism of hydrogen release in real-time. A true comparison of our observations can only be made with similar experimental regimes, namely large samples (1–2 micrometers) and UHV-TEM conditions.

## Summary

Surrey *et al*.^1^ have raised some interesting points. Their interpretation can be expected from experiments using nanocrystalline samples and conventional TEM at low voltages. This was in fact the main contribution of our paper, understanding the dehydriding mechanisms in large samples which was facilitated by *in-situ* viewing using ultra-high voltage TEM. The dependence of dehydriding on sample size and observation conditions highlights the need for our publication. The dehydriding behavior of our material was further confirmed with independent Synchrotron XRD and DSC experiments.

## Additional Information

**How to cite this article:** Nogita, K. *et al*. Reply to ‘Comments on “Evidence of the hydrogen release mechanism in bulk MgH_2_”.’ *Sci. Rep.* **7**, 43720; doi: 10.1038/srep43720 (2017).

**Publisher's note:** Springer Nature remains neutral with regard to jurisdictional claims in published maps and institutional affiliations.
